# Predictors of Discontinued E-Cigarette Use at One-Year Follow-Up in a Sample of Young Adults

**DOI:** 10.3390/ijerph20064770

**Published:** 2023-03-08

**Authors:** Pallav Pokhrel, Crissy T. Kawamoto, Hannah Mettias, Taha Elwir, Thaddeus Herzog

**Affiliations:** Population Sciences in the Pacific Program, University of Hawai‘i Cancer Center, University of Hawai‘i at Mānoa, 701 Ilalo St., Honolulu, HI 96813, USA

**Keywords:** e-cigarette, cessation, abstinence, young adults, predictors, longitudinal

## Abstract

Background: Currently, the research on factors associated with young adults’ discontinuation of e-cigarette use behavior is limited. This study tested the predictors of self-reported e-cigarette abstinence at one-year follow-up among young adult baseline current e-cigarette users. The following variables were tested as predictors: demographics, cigarette smoking, e-cigarette use dependence, e-cigarette use duration, harm perceptions, and preferred aspects of e-cigarette use, including sensations, flavor, and device characteristics. Methods: Data were provided at two time-points one year apart by 435 ethnically diverse young adults (M age = 22.3, SD = 3.1; 63% women) who reported current e-cigarette use at baseline. Results: Approximately 42% of those who reported current e-cigarette use at baseline (i.e., 184 out of 435 participants) reported discontinuation of e-cigarette use at one-year follow-up. Results indicated that higher e-cigarette dependence, longer history of e-cigarette use, lower e-cigarette harm perceptions, greater preference for both menthol and sweet flavors, for open-pod-based devices, and for e-cigarette use sensations such as buzz, taste and smell of flavors, and throat hit at baseline were associated with lower likelihood of e-cigarette use discontinuation at one-year follow-up. Conclusions: Characteristics associated with nicotine (e.g., dependence) and flavors (e.g., taste and smell) appear to drive the continuation/discontinuation of e-cigarette use among young adults. Thus, cessation strategies may need to be developed with a focus on dependence and harm perceptions related to nicotine and flavors. Furthermore, better regulating open-pod-based devices and sweet–menthol flavors may help e-cigarette use prevention.

## 1. Introduction

Among adults in the U.S., young adults (18–25 years old) report the highest prevalence of current e-cigarette use (9.4%) [1]. Recent data show that an increasing proportion of young adult current e-cigarette users have never smoked cigarettes or have never been regular cigarette smokers [2]. E-cigarettes are commonly used by young people for consumption of flavored aerosol [3] that may or may not contain nicotine, and e-cigarette use has been associated with dependence [4,5]. Young adult e-cigarette users tend to report addiction as a concern [6] and face difficulty while trying to quit [7]. There has been an increasing demand for research focused on developing effective strategies for helping e-cigarette users quit e-cigarette use, especially among young users who may not be using e-cigarettes to quit or reduce cigarette smoking [8,9]. The development of effective e-cigarette use cessation strategies requires a greater understanding of the factors associated with the perpetuation of e-cigarette use among young people. Currently, little is known about factors that predict continuation or discontinuation of e-cigarette use among young people over time.

Several young people who experiment with tobacco products, including e-cigarettes, stop using the products after some time [10,11]. Existing findings on predictors of future e-cigarette use cessation among young adults are consistent to the extent that higher nicotine dependence and lower e-cigarette use harm perceptions appear to reduce the likelihood of use discontinuation [8,12,13,14,15,16]. In addition, research tends to show that dual users of cigarettes and e-cigarettes at baseline are less likely to stop using either or both products during study period [16,17]. At present, however, studies have rarely conducted a systematic examination of predictors of e-cigarette use discontinuation, particularly among young adults.

The factors shaping substance use progression among young people are complex and often warrant the consideration of multiple theoretical frameworks [18]. To examine predictors of e-cigarette use discontinuation, the present study considered variables central to social cognitive, dispositional, and affective models of substance use etiology [18] and factors implicated in increase of e-cigarette use dependence and abuse liability [19,20]. Dispositional variables such as sensation seeking have been strongly related to tobacco use progression [21]. Interpersonal influence, which may be assessed in terms of the presence of substance users in one’s social networks, and variables such as harm perceptions and use motives have been central to social cognitive models of tobacco use etiology [18] and have been associated with e-cigarette use [22,23]. Harm perceptions refer to perceived harm related to e-cigarette use and may be assessed with reference to e-cigarette use only (i.e., absolute e-cigarette harm perception) or as compared with cigarette smoking (relative harm perception) [23]. Sensory experiences associated with e-cigarette use such as smell, taste, and throat hit may act as unique motives for e-cigarette use and need to be considered as factors influencing e-cigarette use behavior [24,25]. Indicators of mental health that are related to affect regulation, such as anxiety and depression, have been predictive of continued tobacco use [26] and have been associated with higher e-cigarette use [27]. More specific to e-cigarettes, nicotine concentration and flavor preference have been found to be associated with e-cigarette abuse liability [19,20]. Further, some recent evidence suggests that demographic and device-related characteristics may predict future e-cigarette use discontinuation. Among adolescents, one study [14] found that girls were likely to report lower intentions of quitting e-cigarette use, and that users of cartridge-based pod devices were less likely to quit compared with users of disposable devices. In another study with adult long-term e-cigarette users [16], users of “drip-fed atomizers” were more likely to quit than users of other types of devices. Similar evidence concerning young adults has been lacking.

Thus, the present study has been designed to address existing gaps in the literature related to a systematic examination of predictors of e-cigarette use cessation among young adults. The overall objective of the study is to examine the baseline predictors of future e-cigarette use discontinuation across multiple domains in a sample of young adults. Specifically, predictors across the following domains are examined: socio-demographics (e.g., age, sex, ethnicity), mental health (e.g., anxiety, depression), e-cigarette use history (e.g., duration, dependence), interpersonal influence (e.g., e-cigarette use in social networks), other substance use (e.g., cigarette, marijuana, alcohol), e-cigarette harm perceptions, preferred sensations associated with e-cigarette use (e.g., taste, throat hit), and preferred flavors and device characteristics (e.g., type, nicotine concentration).

## 2. Methods

### 2.1. Participants and Procedures

The present sample represents a subset (i.e., current e-cigarette users) of a larger sample of young adults who participated in a longitudinal study on the effects of e-cigarette marketing on behavior. The procedures followed to recruit and collect data from the larger sample are detailed elsewhere [28]. Briefly, participants were recruited from seven community colleges within the state university system in Hawai‘i. E-mail addresses for all 18- to 29-year-old students enrolled across the colleges were obtained, and an invitation to participate in the study was sent to a random sample of the addresses. However, disproportionately more women who were never cigarette smokers were likely to respond to the email invitation compared with men, cigarette smokers, or e-cigarette users. Thus, in order to recruit more men and cigarette smokers/e-cigarette users, we supplemented the email method of recruitment with classroom-based recruitment.

At each college, approximately 45 classrooms across academic disciplines, including vocational and technical training programs, were randomly selected, and their instructors were approached regarding permission to present study information and collect voluntary paper-and-pencil eligibility screeners. However, we were only able to complete classroom-based recruitment in four out of seven colleges because by the time we finished recruiting at a fourth school, classes had shifted to online instruction in response to the COVID-19 pandemic. Students recruited through e-mail and classroom methods completed the same baseline survey, which was administered online via Qualtrics, which is a web-based survey administration tool. Baseline respondents were contacted again six and twelve months later for follow-up surveys, which were also administered via Qualtrics. The current study involves data collected at baseline and 12-month follow-up. Baseline data were collected between late 2019 and mid-2020; 12-month follow-up data were collected between early to mid-2021.

After providing informed consent, a total of 1604 participants completed the baseline survey, of whom 92% (n = 1469) also completed the 12-month follow-up survey. At baseline, 490 self-identified as current e-cigarette users; of these, 435 also completed the 12-month follow-up survey (89% retention). Thus, the present study is based on 435 baseline current e-cigarette users who provided data at both baseline and 12-month follow-up. For attrition analysis, we compared those lost to follow-up (n = 55) with those retained (n = 435) on baseline data of all study variables. A statistically significant difference was found only for past-30-day cigarette smoking: Those lost to follow-up represented a higher proportion of current cigarette smokers (35%) compared with those retained (22%; *p* = 0.04). The lost and retained samples did not differ on other study variables, including age, sex, weekly work for pay, sensation seeking, cigarette smoking, and marijuana use (*p* > 0.05).

### 2.2. Measures

***Demographics.*** A single item each was used for age, gender, and number of hours worked per week for pay. For ethnicity, participants were asked “What is your ethnic background?” and were provided with a list of ethnicities common in Hawai‘i and the U.S. The question was asked in two different ways: The first question asked participants to refer to the list and “check all that apply”. The second question asked participants to choose the single ethnic background that they identified with most. The response to the second question was utilized to assign mixed-ethnicity individuals to a particular racial/ethnic category.

***Sensation seeking*.** Sensation seeking was assessed with the Brief Sensation Seeking Scale (8 items; α = 0.91, current sample) [29]. Participants were provided statements (e.g., “I like to do frightening things”, “I prefer friends who like to do excitingly unpredictable things”) and asked to rate the extent to which each statement applied to them (5-point scale ranging from “Never” to “Usually”).

***Social network e-cigarette use.*** Characteristics of participants’ in-person or “offline” social networks (i.e., as opposed to virtual or online social networks) were assessed using the egocentric method [30]. Participants were asked to nominate up to 10 individuals whom they spend the most time with or talk to most often. Next, they were asked a number of questions on each of the individuals that they nominated. One of the questions was on e-cigarette use: “Does this person use e-cigarettes?” (Response options: “No, not at all”, “Yes, sometimes”, and “Yes, regularly”). For analysis, an index for the presence of e-cigarette users in social networks was created by summing up any “yes” response across the nominees.

***Anxiety*.** The brief Generalized Anxiety Disorder Scale (7 items; GAD-7) [31] was used. Participants were asked to indicate how often they experienced certain anxiety symptoms in the past week (4-point scale ranging from “Not at all” to “Nearly every day”; α = 0.93, current sample).

***Depression*.** Depression was assessed using the Center for Epidemiological Studies Depression (CES-D) Scale [32]. The scale includes 20 items, which ask questions about experience with depressive symptoms in the past week (4-point scale ranging from “Less than 1 day” to “5–7 days”; α = 0.90, current sample).

***E-cigarette use duration*.** E-cigarette use history was assessed with a single item, “How long have you been using an e-cigarette? (5-point scale; “Less than a month”, “1–6 months”, “Less than a year”, “1–2 years”, “2–5 years”, “Over 5 years”).

***E-cigarette harm perceptions*.** Harm perceptions related to e-cigarettes were assessed in terms of both harm perceptions compared with cigarette smoking (i.e., relative harm perceptions) and harm perceptions regarding e-cigarettes solely (i.e., absolute harm perceptions). Relative harm perceptions were assessed with a 20-item measure (α = 0.94, current sample) used successfully in previous research [6,23]. Example items include, “E-cigarettes provide safer way to get nicotine” and “E-cigarettes are less addictive than cigarettes” (7-point scale, ranging from “Strongly Disagree” to “Strongly Agree”). Absolute harm perceptions were measured using 8 items (α = 0.85, current sample). Example items include “Vaping is unhealthy” and “Vaping is addictive” (7-point scale, ranging from “Strongly Disagree” to “Strongly Agree”).

***E-cigarette use dependence*.** E-cigarette use dependence was assessed using the 10-item Penn State E-cigarette Dependence Index [33] (α = 0.90, current sample).

***E-cigarette use sensations*.** Preferred e-cigarette use sensations were assessed based on six items adapted from the Sensory E-cigarette Expectancies Scale (SEES) [25]. Participants were asked, “People enjoy various feelings, sensations, and activities associated with e-cigarette use, which makes them want to continue using e-cigarettes. How much do the following matter to you as an e-cigarette user?” Each sensation measuring taste, smell, buzz, throat hit, vape-tricks, or tinkering with devices was scored on a 10-point scale ranging from 0 (“Not at all”) to 9 (“A lot”). Each sensation was tested as an independent variable in the current study.

***Flavors*.** To assess flavor preference, participants were asked, “What flavor e-liquid do you use most commonly?” with an instruction to check all that applied from the list of flavor types provided: fruit (e.g., apple, watermelon), candy (e.g., cotton candy, bubble gum), dessert (e.g., vanilla cream, pineapple sherbet), cereal (e.g., Fruity Pebbles, Frosted Flakes), menthol (e.g., menthol, mint, spearmint), tobacco, marijuana, and alcoholic (pina colada, mojito) and non-alcoholic (e.g., fruit punch, milk tea boba) beverages. For analysis purposes, flavor preference was separated into the following categories: sweet only, sweet and menthol, and other (i.e., tobacco, marijuana, and menthol-only).

***Devices*.** Participants were asked, “How often do you use the following types of e-cigarette device?” A list of device types, each accompanied by example images, was provided: “Cig-a-like”, “Ego-style tank system”, “Mod or an advanced personalized vaporizer (AVP)”, “JUUL or a similar pod-based device”, and “Suorin or similar open pod devices.” Frequency of use was assessed on a 5-point scale (“I have never used this type of e-cigarette”, “I used to use this type but not any longer”, “Rarely”, “Sometimes”, and “Usually”). An open-ended “Other” option was provided to obtain information on types of devices preferred other than the devices provided in the list.

***Nicotine concentration*.** Participants were asked, “What is the nicotine concentration of the e-liquid (e-juice) that you use most frequently?” Options provided were, “I don’t know”, “0 mg/mL”, “1–4 mg/mL”, “5–8 mg/mL”, “9–16 mg/mL”, “17–24 mg/mL”, and “Over 24 mg/mL.”

***E-cigarette use*.** Two measures of current use were utilized. The first item asked participants, “How often, if at all, do you currently use an e-cigarette or a similar vaping device?” (Response options: “Daily”, “Less than daily, but at least once a week”, “Less than weekly, but at least once a month”, “Less than monthly”, and “Not at all”) [34]. The second item assessed current use in terms of past-30-day use: “Within the past 30 days (1 month), on how many days did you use an e-cigarette or a similar vaping device?” (Response options: “0 days”, “1–2 days”, “3–5 days”, “6–9 days”, “10–19 days”, “20–29 days”, and “All 30 days”).

***Cigarette smoking*.** Current cigarette smoking was assessed in terms of past-30-day use: “Within the past 30 days (1 month), on how many days did you use a cigarette?” (Response options: “0 days”, “1–2 days”, “3–5 days”, “6–9 days”, “10–19 days”, “20–29 days”, and “All 30 days”).

***Marijuana use*.** Current marijuana use was assessed in terms of past-30-day use in two ways. We asked a question about general marijuana use, “Within the past 30 days (1 month), on how many days did you use marijuana (cannabis)?” (Response options: “0 days”, “1–2 days”, “3–5 days”, “6–9 days”, “10–19 days”, “20–29 days”, and “All 30 days”). We also asked a question about marijuana vaping: “Within the past 30 days (1 month), on how many days did you vape marijuana using an e-cigarette or a similar vaping device?” Both items were considered to create a categorical variable of marijuana use representing non-use, use by vaping, and use by other means.

***Binge drinking*.** Participants were asked, “Over the last two weeks, how many times have you had 5 or more drinks of alcohol at one sitting?” Response was scored on a 10-point scale ranging from “0 times” to “10 times or more.”

### 2.3. Statistical Analysis

Statistical analyses were performed in SAS (Version 9.3) using multiple logistic regression. Two multiple regression models were run. Model 1 tested the effects of baseline e-cigarette use dependence, sensation seeking, past-30-day cigarette smoking, and demographic variables (i.e., age, sex, ethnicity, hours worked per week for pay), simultaneously, on e-cigarette use discontinuation at one-year follow-up. Model 2 tested the effects of other baseline predictor variables (i.e., social network e-cigarette use, anxiety, depression, harm perceptions, alcohol and marijuana use, preferred sensations, and device characteristics) separately, adjusting for all Model 1 independent variables. Since participants were recruited from seven colleges, we examined intra-class correlations for e-cigarette use and cigarette smoking among participants by college to get a sense of whether college-level random effects needed to be accounted for in the regression analysis. We found the intra-class correlation coefficient (<0.02) to be negligible, which is understandable given that community college students are commuters and may be less susceptible to college-level characteristics. Hence, the present logistic regression models did not account for college-level random effects. Missing data across variables were close to 5%. We used listwise deletion to address missing data.

## 3. Results

### 3.1. Participant Characteristics

Participants were young adults who self-identified as current e-cigarette users at baseline and completed a 12-month assessment (N = 435) (see Measures above for the assessment questions completed by the participants). The response rate for the e-mail-based recruitment was 58%. The response rate for classroom-based recruitment was 82%. Table 1 shows the participants’ demographic characteristics. Participants were 18–29 years old and represented more women than men. The sample was multiethnic, with the majority identifying as East Asian, Filipino, or Native Hawaiian/Other Pacific Islander. Twenty-two percent of the sample reported having also smoked a combustible cigarette in the past 30 days.

### 3.2. E-Cigarette Use Characteristics

Table 1 shows the descriptives for participants’ baseline e-cigarette use characteristics. Two self-reported measures were used to ascertain participants’ e-cigarette use status: The first measure asked participants to report their current e-cigarette use status and included an option for “less than monthly” use; the second measure asked participants for their past-30-day e-cigarette use frequency. Participants who reported any use in either of the measures were considered users. Discontinued use was assessed in terms of self-reported non-use across both measures. The majority of the participants were daily e-cigarette users and had been using e-cigarettes for more than six months. Most of the participants who used e-cigarettes did so exclusively (i.e., not dual users). Participants tended to prefer mods or pod-based devices, and the majority preferred sweet-only or a combination of sweet and menthol flavors. Approximately a quarter of the sample reported not knowing the nicotine concentration of the e-liquid they consumed most commonly. In terms of sensations associated with e-cigarette use, participants expressed preferences for the “buzz”, and taste and smell, over other characteristics. Approximately 42% of those who reported current e-cigarette use at baseline (i.e., 184 out of 435 participants) reported discontinuation of e-cigarette use at one-year follow-up, deduced based on two measures of current e-cigarette use: past-30-day e-cigarette use and the measure that took into consideration current use that was less frequently than monthly.

### 3.3. Regression Analyses

Table 2 shows the results of the regression analyses. Higher e-cigarette dependence at baseline was associated with decreased odds of e-cigarette use discontinuation at one-year follow-up, adjusting for sensation seeking, past-30-day cigarette smoking, age, sex, ethnicity, and hours worked per week for pay. A one-unit increase in e-cigarette dependence at baseline reduced the odds of e-cigarette use discontinuation at one-year follow-up by 17%. Baseline sensation seeking, past-30-day cigarette smoking, age, sex, ethnicity, and hours worked per week for pay were not found to be associated with a likelihood of e-cigarette discontinuation at one-year follow-up.

Adjusting for baseline e-cigarette dependence, cigarette smoking, sensation seeking, and demographic variables, length of e-cigarette use duration and lower perceived harms associated with e-cigarette use at baseline were associated with decreased odds of e-cigarette use discontinuation at one-year follow-up. Similarly, higher preference for “buzz”, taste and smell, and “throat hit” as sensations associated with e-cigarette use at baseline were associated with reduced likelihood of e-cigarette use discontinuation at follow-up. Adjusting for baseline e-cigarette dependence, cigarette smoking, sensation seeking, and demographic variables, preference for sweet and menthol flavors (relative to sweet only), use of open-pod-based devices, and higher nicotine concentration e-liquid use were associated with decreased odds of e-cigarette use discontinuation at follow-up.

After adjusting for baseline e-cigarette use dependence, sensation seeking, cigarette smoking, and demographic variables, we did not find an association between either baseline marijuana use (including marijuana vaping) or binge drinking and 12-month e-cigarette use discontinuation. Likewise, no association was found between baseline anxiety, depression, and follow-up e-cigarette use discontinuation.

## 4. Discussion

To our knowledge, this is one of the first studies to examine predictors of e-cigarette use discontinuation among young adults using actual longitudinal changes in use behavior over the course of a year. The data indicated several new findings and replicated some of the previous findings reported in the literature. Notably, consistent with the existing literature, we found that higher e-cigarette use dependence and lower e-cigarette harm perceptions were strongly associated with lower likelihood of e-cigarette use discontinuation at one-year follow-up. A novel aspect of the current research is that both relative and absolute harm perceptions were tested as predictors of e-cigarette use discontinuation: The finding that both types of harm perceptions may be significant predictors of e-cigarette use discontinuation suggests the need to incorporate both types in development of cessation and prevention programs.

Another novel aspect of the current research is that we examined several baseline e-cigarette use characteristics—namely, e-cigarette use duration, preferred device types, flavors, sensations, and nicotine concentration—as predictors of e-cigarette use discontinuation at one year, after adjusting for baseline e-cigarette use dependence, sensation seeking tendencies, and demographic variables. A finding in this domain that may necessitate further research is the inverse association between preference for open-pod-based devices and e-cigarette use dependence. To our knowledge, the only other study [16] that examined preferred device type as a prospective predictor of e-cigarette quitting behavior found the use of drip-based atomizers to predict cessation; however, the Sobieski study was based on adults who had been long-term e-cigarette users. As suggested by some recent evidence [35], with flavor restrictions already taking place on closed-pod-based devices (i.e., devices that use prefilled cartridges) such as JUUL, open-pod-based devices, which are refillable and customizable, may be being used more commonly by more serious e-cigarette users among young people. In general, pod-based devices that use salt-based nicotine are known to deliver nicotine more efficiently [36]. Regulatory authorities may benefit from paying closer attention to open-pod-based e-cigarette devices.

The finding of the inverse effects of preference for both sweet and menthol flavors on e-cigarette use discontinuation is consistent with the high appeal of combined sweet and menthol flavors, or “ice-hybrid” flavors among young users [35,37]. Ice-hybrid flavors appear to better mask the harshness of nicotine and enhance the perceived pleasantness of sweet flavors, thus increasing the potential for abuse and dependence [37]. Hence, policies to better regulate sweet–menthol flavors may be needed.

To our knowledge, this is also the first study to examine preferred sensations or activities associated with e-cigarette use as prospective predictors of e-cigarette use discontinuation. We did not find activities common among e-cigarette hobbyists such as engaging in vape tricks or tinkering with devices [38] to be significant predictors of e-cigarette use discontinuation. On the other hand, preferred sensations such as buzz from nicotine, pleasant taste and smell associated with flavors, and throat hit were significant predictors of lower likelihood of e-cigarette use cessation. These findings, together with findings related to harm perceptions, e-cigarette dependence, nicotine concentration, open pod devices, and ice-hybrid flavors, indicate that continued e-cigarette use among young adults may be driven primarily by nicotine, flavors, and lower perceived harm associated with e-cigarette use. E-cigarette cessation use programs may benefit from addressing e-cigarette-related harm perceptions and by focusing on nicotine and flavor dependence.

Although prior studies have found associations between e-cigarette use and poor mental health [39], we did not find depression or anxiety to be associated with e-cigarette use discontinuation in our sample of young adults. Similarly, the current data did not indicate that cigarette smoking at baseline (i.e., dual use) was associated with e-cigarette use discontinuation at follow-up. This latter finding contradicts other research [16,17]. A possible explanation could be that dual use of cigarettes and e-cigarettes was not highly prevalent in the current sample: Only 22% of the baseline sample reported current cigarette smoking in addition to e-cigarette use. National data indicate that among adolescent current e-cigarette users, dual use decreased significantly between 2014 and 2019 [14], and it is possible that this trend is reflected in young adults as well. The present data may be more recent than most data currently reported in the published literature and may better represent the tendencies among young adults who are exclusive e-cigarette users, though more research is needed to replicate the current findings.

There are limitations to the current study. The study was based on self-reported behavior, which is subject to recall bias. The sample was recruited from community colleges in Hawai‘i, represented more women than men, and was predominantly Asian/Pacific Islander (API). Although the focus of the current research was not culture or gender specific, there may be some concerns about the generalizability of the findings with respect to the U.S. at large. Secondly, despite the fact that the number of participants lost to follow-up was small, those who were lost to follow-up were more likely to represent current cigarette smokers than those who were retained. This may have affected our findings related to the effects of dual use on e-cigarette use discontinuation, even though only mildly. Thirdly, the current data, including information on e-cigarette use discontinuation, were entirely based on self-reports. Although we used two separate measures to confirm e-cigarette use and non-use, we did not bio-verify use and non-use. Lastly, the current study did not assess the use of pod-based disposable e-cigarettes. Thus, given these limitations, there is a need to replicate the present findings or pursue similar questions in other populations and using additional measurement instruments.

## 5. Conclusions

Despite these limitations, the present study elucidates key potential predictors of e-cigarette use continuation/discontinuation among young adults. A major implication of the study is that continued e-cigarette use appears to be driven by nicotine and flavors, and, thus, cessation strategies may need to be developed with a focus on dependence and harm perceptions related to nicotine and flavors. Furthermore, stronger regulations on open-pod-based devices and sweet–menthol flavors may help e-cigarette use prevention. In conclusion, the present study indicates that higher e-cigarette dependence, greater preference for sweet and menthol flavors, greater preference for sensations such as throat hit, smell, and taste, and preference for higher nicotine continuation are associated with lower likelihood of discontinued e-cigarette use among young adults.

## Figures and Tables

**Table 1:** Baseline characteristics of the longitudinal sample (N = 435).

	Mean (SD)	Frequency (n)	Range
Age	22.3 (3.1)		18–30
Sex			
Men		37% (159)	
Women		63% (276)	
Ethnicity			
East Asian		19% (83)	
Filipino		26% (114)	
Native Hawaiian		20% (85)	
Other		6% (27)	
White		29% (126)	
Hours worked for pay per week			
0 h		18% (80)	
1–9 h		10% (41)	
10–19 h		20% (85)	
20–29 h		25% (109)	
30–39 h		12% (54)	
40 h		10% (43)	
More than 40 h		5% (22)	
E-cigarette use frequency ^1^			
Less than monthly		16% (71)	
Monthly but less than weekly		32% (139)	
Weekly but less than daily		15% (67)	
Daily		36% (158)	
E-cigarette use duration			
Less than 1 month		14% (56)	
1–6 months		10% (43)	
Less than 1 year		10% (43)	
1–2 years		31% (127)	
2–5 years		28% (115)	
More than 5 years		7% (28)	
E-cigarette harm perceptions ^2^			
Low harm perception (absolute)	3.5 (1.3)		1–7
Low harm perception (relative to cigarettes)	3.8 (1.4)		1–7
E-cigarette use dependence ^3^	4.8 (4.7)		0–19
Past-30-day cigarette smoking			
No		78% (339)	
Yes		22% (96)	
Past-30-day marijuana use			
None		56% (243)	
Vaped		28% (121)	
Other only		16% (71)	
Past-2-week binge drinking			
No		52% (228)	
Yes		48% (207)	
Preferred flavor			
Sweet only		41% (178)	
Sweet and menthol		38% (164)	
Other		21% (93)	
Preferred device			
Cigalike	1.4 (0.83)		1–5
Tank system	1.9 (1.0)		1–5
Mods	2.7 (1.3)		1–5
Juul or other closed pods	2.4 (1.4)		1–5
Suorin or other open pods	2.6 (1.5)		1–5
Preferred nicotine concentration			
0 mg/mL		16% (54)	
1–4 mg/mL		32% (107)	
5–8 mg/mL		20% (66)	
9–16 mg/mL		3% (10)	
17–24 mg/mL		4% (13)	
Over 24 mg/mL		25% (83)	
Preferred sensations			
Buzz		5.8 (3.2)	1–10
Flavor smell		6.0 (2.9)	1–10
Flavor taste		6.7 (2.8)	1–10
Throat hit		4.2 (3.0)	1–10
Vape tricks such as blowing clouds		3.7 (3.1)	1–10
Tinkering with device		2.4 (2.3)	1–10
Sensation seeking	3.0 (0.94)		1–5
Social network e-cigarette use	3.3 (2.4)		0–10
Anxiety	2.0 (0.83)		1–4
Depression	1.9 (0.57)		1–4

Note: SD = standard deviation. ^1^ Categorization took into consideration both measures of current e-cigarette use (see measures). ^2^ Relative harm perceptions refer to perceived harm due to e-cigarette use relative to cigarette smoking and absolute harm perceptions refer to perceived harm related to e-cigarette use itself. ^3^ E-cigarette dependence refers to a construct representing physical dependence on e-cigarette use.

**Table 2:** Baseline predictors of discontinued e-cigarette use one year later (N = 435).

Baseline Predictors		Discontinued Use at 1-Year Follow-Up ^1^	
		Odds Ratio (95% CI)	*p*-Value
Model 1 ^2^			
E-cigarette dependence		0.83 (0.78–0.87)	<0.0001
Sensation seeking		0.99 (0.96–1.02)	0.34
Past-30-day cigarette use			
	No	1	
	Yes	0.80 (0.48–1.38)	0.41
Age		1.07 (0.99–1.14)	0.09
Sex	Men	1	
	Women	1.14 (0.74–1.75)	0.56
Ethnicity	White	1	
	East Asian	1.04 (0.57–1.89)	0.85
	Filipino	0.88 (0.51–1.54)	0.55
	NHPI	1.09 (0.59–2.00)	0.69
	Other	0.98 (0.40–2.40)	0.98
Hours worked		0.90 (0.79–1.02)	0.09
Model 2 ^3^			
Social network e-cigarette use		0.90 (0.82–0.99)	0.02
Anxiety		1.15 (0.89–1.49)	0.33
Depression		1.16 (0.81–1.68)	0.42
E-cigarette use duration		0.81 (0.70–0.93)	0.004
Low harm perception (absolute) ^4^		0.71 (0.60–0.85)	0.0001
Low harm perception (relative) ^4^		0.65 (0.55–0.78)	<0.0001
Preferred sensations	Buzz	0.91 (0.85–0.98)	0.009
	Flavor smell	0.90 (0.83–0.97)	0.006
	Flavor taste	0.87 (0.80–0.94)	0.0003
	Throat hit	0.89 (0.83–0.96)	0.004
	Vape tricks	1.04 (0.97–1.12)	0.32
	Tinkering with device	0.96 (0.88–1.06)	0.42
Past-30-day marijuana use			
	None	1	
	Vaped	1.36 (0.83–2.24)	0.26
	Other form only	1.04 (0.58–1.89)	0.70
Past-2-week binge drinking	No	1	
	Yes	0.68 (0.45, 1.05)	0.08
Preferred flavor			
	Sweet only	1	
	Sweet and menthol	0.43 (0.26–0.69)	0.0001
	Other	1.08 (0.62–1.89)	
Preferred device			
	Cigalike	1.17 (0.90–1.53)	0.24
	Tank system	1.08 (0.88–1.33)	0.45
	Mods	0.92 (0.78–1.09)	0.35
	Juul or other closed pods	1.02 (0.87–1.20)	0.82
	Suorin or other open pods	0.76 (0.64–0.89)	0.001
Preferred nicotine concentration		0.76 (0.65–0.89)	0.0007
Knowledge of nicotine concentration used			
	Yes	1	
	No	1.36 (0.82–2.24)	0.23

Notes: CI: confidence interval. ^1^ Discontinued use was coded as 1: discontinued use, 0: continued use; ^2^ all the variables in Model 1 were entered in the regression model simultaneously as independent variables. ^3^ Each variable listed under Model 2 was tested separately as an independent variable, adjusting for all independent variables included in Model 1. ^4^ Higher values indicate lower e-cigarette harm perceptions.

## Data Availability

Data may be made available upon formal request. Please submit any such request to the first author (ppokhrel@cc.hawiaii.edu).
